# Effect of pH-sensitive nanoparticles on inhibiting oral biofilms

**DOI:** 10.1080/10717544.2022.2037788

**Published:** 2022-02-14

**Authors:** Xinyu Peng, Qi Han, Xuedong Zhou, Yanyan Chen, Xiaoyu Huang, Xiao Guo, Ruiting Peng, Haohao Wang, Xian Peng, Lei Cheng

**Affiliations:** a State Key Laboratory of Oral Diseases, West China Hospital of Stomatology, National Clinical Research Center for Oral Diseases, Sichuan University, Chengdu, China; b Department of Operative Dentistry and Endodontics, West China School of Stomatology, Sichuan University, Chengdu, China; c Department of Oral Pathology, West China School of Stomatology, Sichuan University, Chengdu, China

**Keywords:** pH-sensitive drug delivery system, nanocarrier, chlorhexidine, dental caries, *S. mutans*, intelligent material

## Abstract

Dental caries is a biofilm-related preventable infectious disease caused by interactions between the oral bacteria and the host’s dietary sugars. As the microenvironments in cariogenic biofilms are often acidic, pH-sensitive drug delivery systems have become innovative materials for dental caries prevention in recent years. In the present study, poly(DMAEMA-co-HEMA) was used as a pH-sensitive carrier to synthesize a chlorhexidine (CHX)-loaded nanomaterial (p(DH)@CHX). *In vitro*, p(DH)@CHX exhibited good pH sensitivity and a sustained and high CHX release rate in the acidic environment. It also exhibited lower cytotoxicity against human oral keratinocytes (HOKs) compared to free CHX. Besides, compared with free CHX, p(DH)@CHX showed the same antibacterial effects on *S. mutans* biofilms. In addition, it had no effect on eradicating healthy saliva-derived biofilm, while free CHX exhibited an inhibitory effect. Furthermore, the 16s rDNA sequencing results showed that p(DH)@CHX had the potential to alter oral microbiota composition and possibly reduce caries risk. In conclusion, the present study presents an alternative option to design an intelligent material to prevent and treat dental caries.

## 1. Introduction

Dental caries is associated with orofacial pain and, when untreated, can lead to tooth loss and systemic infection (Kabani et al., 2020; Liang et al., 2020). Dental caries is a dynamic pathological process dependent on the presence of complex polymicrobial biofilms known as dental plaque (Takahashi & Nyvad, 2011). When the microbial ecological balance is disrupted, pathogenic bacteria produce acid from food particles aggregated on the tooth surface through a fermentation process, promoting tooth demineralization (Simon-Soro & Mira, 2015; Hajishengallis et al., 2017). *Streptococcus mutans*, considered the major etiologic factor of dental caries, is an opportunistic oral pathogen that resides in the multispecies biofilm (Kuramitsu, 1993). It can rapidly colonize tooth surfaces and establish cariogenic biofilms with extracellular polysaccharides (EPS) (Guo et al., 2016; Wang & Ren, 2017). This bacterial species can ferment sugars to produce acid and acidify the local microenvironment (Hamada & Slade, 1980). As a result, there is a steep fall in pH in dental plaque, leading to the demineralization of the tooth enamel and the development of tooth decay (Khara et al., 2018). Previous research has proved that the pH at active caries sites can be approximately 4.5–5.5 (Bowen, 2013). Therefore, it is crucial to prevent and treat early carious lesions.

However, the prevention of dental caries is associated with serious challenges. Conventional medicines have exhibited several side effects (Featherstone, 2006; Gadallah et al., 2018). For example, chlorhexidine (CHX) is a cationic charged broad-spectrum antibacterial agent widely used in dental clinical practice and is the gold standard against dental biofilms (Jones, 1997). Also, CHX can inhibit the catalytic activity of matrix metalloproteinases (MMPs) (Zhou et al., 2016). Nevertheless, continuous use of CHX has several disadvantages, such as pronounced cytotoxicity *in vitro* (Pucher & Daniel, 1992; Babich et al., 1995) and no modulatory effects on oral biofilms. Thus, CHX is not recommended for long-term therapeutic use (Flötra et al., 1971). Efforts have been made to develop novel anti-cariogenic materials to deal with these issues. In recent decades, with the development of nanotechnology, novel stimuli-responsive materials have been widely applied and well developed in the field of biomedicine. Since the 1970s, with an increased understanding of pathophysiology, different pH-sensitive polymers have been used to develop smart materials that could potentially help treat various diseases (Yatvin et al., 1980). Nowadays, these intelligent materials are widely used to treat different diseases, including cancer, cardiovascular diseases, hypertension, peptic ulcers, etc. (Balamurali et al., 2010). Compared with traditional pharmaceutical treatment modalities, they are effective in specific targeting and controlled release, with lower toxicity and better bioavailability (Makowski et al., 2019). The human body’s pathologic environment, such as tumor tissues and inflammatory sites, is more acidic than normal healthy parts (Gumustas et al., 2016; Li et al., 2017). Similarly, the microenvironment of dental caries is different from that of the healthy oral cavity (Zhao et al., 2019). As described before, the pH of the local micro-ecological environment of dental caries is reduced to 4.5–5.5, while in the physiological environment, saliva has a normal pH range of 6.2–7.6, with 6.7 being the average pH (Baliga et al., 2013). Therefore, pH-sensitive smart materials can prevent dental caries. Some researchers used novel antimicrobial agents to develop innovative caries-preventing materials. Some researchers designed farnesol-loaded pH-sensitive nanoparticles to disrupt oral biofilm virulence (Horev et al., 2015). These nanoparticles exhibited great adsorption affinity for negatively charged HA, sHA, and exopolysaccharide-coated sHA. Farnesol, an antibacterial agent with limited antibiofilm and anti-cariogenic effects, became an effective therapy against dental caries after being loaded into the nanoparticles. In addition, dextran-coated iron oxide nanoparticles, termed nanozymes, with vigorous catalytic activity at acidic pH values and high specificity on targeted biofilms, were synthesized by the same group (Naha et al., 2019). Also, they could prevent severe dental caries without impacting surrounding oral tissues *in vivo*. Moreover, other researchers reported tertiary amine-modified resin adhesives (TA@RAs), with pH-responsive antibacterial effect, as novel intelligent materials to reduce the occurrence of secondary dental caries (Liang et al., 2020). Furthermore, other researchers incorporated traditional antimicrobial agents into pH-responsive carriers to overcome the drawbacks of these conventional drugs. For example, some researchers designed a pH-responsive nanocarrier system capable of releasing CHX in acidic milieus within cariogenic biofilms (Zhao et al., 2019). Furthermore, some other researchers fabricated pH- and glutathione-responsive, biodegradable disulfide-bridged mesoporous silica nanoparticles (MSNs) to co-deliver silver nanoparticles and CHX for biofilm inhibition (Lu et al., 2018). Both studies demonstrated that smart drug delivery systems could reduce the toxicity of CHX and exhibit outstanding antibacterial properties in *S. mutans* biofilms. However, they only reported the antibacterial efficacy of delivered CHX without exploring its micro-ecosystem-regulating capabilities.

Inspired by the specific properties of dental caries, we prepared pH-sensitive nanoparticles, referred to as poly(N,N-dimethylaminoethyl methacrylate(DMAEMA)-co-2-hydroxyethyl methacrylate(HEMA)) nanoparticles, for the triggered release of CHX within a caries microenvironment. DMAEMA is a cationic monomer with a pKa around 7.5 that undergoes structural changes below its pKa (Van De Wetering et al., 1999). Therefore, polymers containing DMAEMA undergo structural changes (swelling) due to the protonation of DMAEMA amine groups in acidic environments (Brahim et al., 2003). As a hydrophilic monomer, HEMA is a candidate in the synthesis of nanohydrogels or induction of hydrophilicity in hydrophobic surfaces (González-Henríquez et al., 2019). HEMA has commonly been used as the carrier for drug delivery applications to treat various diseases (Gulsen & Chauhan, 2005; You & Auguste, 2008; Roointan et al., 2018). In the present report, researchers studied the carrier’s properties at different pH values and its ability to target cariogenic biofilms. It indicates that poly(DMAEMA-co-HEMA) might prevent dental caries, as it could fit the characteristics of the oral microenvironment to release antibacterial drugs only at the caries site to avoid disrupting the biofilm at the non-caries site; at the same time, it could reduce the cytotoxicity of CHX and the possibility of ecologic imbalance.

## 2. Materials and methods

### 2.1. Synthesis and characterization of poly(DMAEMA-co-HEMA)

Triethylene glycol dimethacrylate (TEGDMA) was obtained from Nippon Shokuba (Osaka, Japan). Other materials like (dimethylamino)ethyl methacrylate (DMAEMA), co-monomer 2-hydroxyethyl methacrylate (HEMA), 2-dimethylamino-4-(methyl-phenylamino)-phenol (DMPAP), and CHX were obtained from Sigma and were used as received, unless otherwise stated.

**Figure 1.**
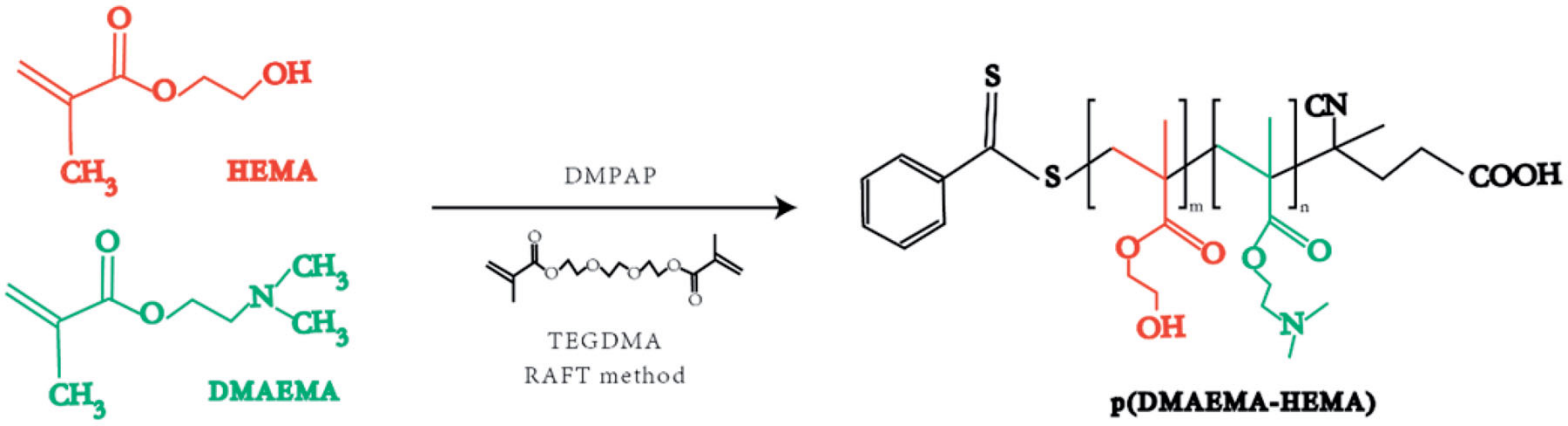
Fabrication of poly(DMAEMA-co-HEMA).

Polymers were obtained through the atom transfer radical polymerization (ATRP) synthesis, as previously reported (Roointan et al., 2018). As presented in Figure 1, equimolar amounts of water and glycol were used as the solvent for preparing the pre-cross-linked hydrogels. The monomer 2-(dimethylamino)ethyl methacrylate (DMAEMA) and co-monomer 2-hydroxyethyl methacrylate (HEMA) were mixed at a ratio of 3:1. Afterward, the reaction was induced by adding the photoinitiator DMPAP and the cross-linker TEGDMA. Finally, poly(DMAEMA-co-HEMA) was obtained after purification. The FT-IR spectra of the polymer were obtained using an FT-IR spectrometer (IRTracer-100, SHIMADZU, Kyoto, Japan). The size and morphology of DMAEMA/HEMA nanoparticles were analyzed by dynamic light scattering (Zetasizer Nano ZS, MALVERN, Worcestershire, UK) and transmission electron microscopy (Tecnai G2 F20 S-TWIN, FEI, Hillsboro, OR). To obtain the size of poly(DMAEMA-co-HEMA) in different pH values, 1 mg/mL of poly(DMAEMA-co-HEMA) nanoparticles in an aqueous solution was measured at different pH values in phosphate-buffered solution (PBS) buffer (pH values of 3, 4, 5, 6, 7, 8, 9, and 10). After preparing the solutions with different pH values, the nanoparticle was dispersed using a magnetic stirrer for almost one hour. In further analysis, particle sizes were measured by the dynamic light scattering (DLS) method. To obtain transmission electron microscope (TEM) images, 1 mg/mL of poly(DMAEMA-co-HEMA) nanoparticles at different pH values (4.5 and 7) was dropped onto a copper grid supporting a thin film of amorphous carbon. All the measurements were performed three times and the average values were reported.

### 2.2. Drug loading and *in vitro* CHX release from p(DH)@CHX

To obtain p(DH)@CHX, 7.5 mg of CHX was added to 3 mL of poly(DMAEMA-co-HEMA) solution (5 mg/mL), and the mixture was stirred at room temperature for two hours. CHX-loaded poly(DMAEMA-co-HEMA) was washed with deionized water, and the volume of CHX was measured by HPLC (Mai et al., 2019). The loading efficiency was determined using Equation (1), the drug-loading content was calculated according to Equation (2) (Wang et al., 2020).

(1) $$\text{Encapsulation}\;\text{efficiency}\;(\%)=\frac{\text{the}\;\text{amount}\;\text{of}\;\text{drug}\;\text{loaded}}{\text{the}\;\text{amount}\;\text{of}\;\text{drug}\;\text{before}\;\text{loading}}\times 100\%$$

(2) $$\text{Drug}-\text{loading}\;\text{content}\;(\%)=\frac{\text{the}\;\text{amount}\;\text{of}\;\text{drug}\;\text{loaded}}{\text{the}\;\text{amount}\;\text{of}\;\text{the}\;\text{carrier}}\times 100\%$$

To investigate the release behavior of CHX, 1 mL p(DH)@CHX (12.5 mg/mL) was dispersed in PBS solutions (a pH value of 7 or 4.5). Then, the mixture was transferred into a dialysis bag (molecular weight cutoff = 500) on a shaking table at 37 °C for one hour. At each time interval (0, 20, 40, and 60 minutes), the release medium was withdrawn from the centrifugal tubes and replaced with a freshly released medium to maintain the sink condition (Liang et al., 2017). The cumulative amount of CHX released was determined by HPLC.

### 2.3. Cytotoxicity assessment of poly(DMAEMA-co-HEMA) and p(DH)@CHX

The *in vitro* cytotoxicity of poly(DMAEMA-co-HEMA), CHX, and p(DH)@CHX was evaluated by CCK-8 assay using human oral keratinocyte (HOK) cell line (JENNIO, Guangzhou, China). In brief, HOK cells (8 × 10^3^ cells/well) were seeded in a 96-well microplate and incubated for 24 hours. Subsequently, the culture media were replaced by fresh culture media containing serial dilutions for further cultivation of 24 hours. Then, CCK-8 solution was added to each well and incubated at 37 °C for one hour. Finally, the absorption was measured by a microplate reader at 450 nm (Zhou et al., 2019).

### 2.4. Antibacterial assays

#### 2.4.1. Bacterial strains

Fifteen microliters of stock *S. mutans* bacteria (ATCC UA159; American Type Culture Collection, Manassas, VA) were added to 15 mL of brain heart infusion (BHI) broth (Becton, Dickinson and Company, Franklin Lakes, NJ) and incubated at 37 °C with 5% CO_2_ for 16 hours. In addition, the biofilms were incubated on glass disks in BHI broth supplemented with 1% sucrose (BHIS) (Solarbio, Beijing, CN). For the biofilm inoculation, *S. mutans* suspension was diluted 10 folds in BHIS to form the inoculation medium. The glass disks were placed in the wells of a 24-well plate, and 1.5 mL of the inoculation medium was seeded into each well. Then, the plate was incubated at 5% CO_2_ and 37 °C for 24 hours to form biofilms (Cheng et al., 2011).

#### 2.4.2. Determination of the minimum inhibitory concentration (MIC) and minimum bactericidal concentration (MBC)

To evaluate the antibacterial properties of p(DH)@CHX and CHX, a microtiter plate assay was used to determine the MIC and MBC for *S. mutans*, following a previous method (Li et al., 2013). MIC measurements relied on the serial twofold microdilution method using BHI broth. p(DH)@CHX at 1.25 mg/mL, poly(DMAEMA-co-HEMA) at 1.25 mg/mL, and CHX at 100 μg/mL served as the starting concentrations. Overnight cultures of bacteria in the broth were diluted by fresh medium and used for the experiments. The bacterial culture was adjusted to 2 × 10^6^ CFU/mL of suspension, and 100 μL was inoculated into each well in the 96-well plate with the culture medium. After incubation at 37 °C with 5% CO_2_ for one day, MIC was determined by visual examination: the lowest concentration at which no visible bacterial growth appeared was recorded as the MIC. All the tests were carried out three times each time and then repeated three times on different days.

MBC was determined as the lowest concentration of the antimicrobial agent that killed 99.9% of the initial inoculum. Ten microliters of aliquots of bacterial suspensions from the wells without viable cells, where bacterial growth was inhibited, were inoculated onto the surface of BHI agar plates and incubated under anaerobic conditions for 48 hours at 37 °C with 5% CO_2_. After calculating the colony-forming units (CFUs), the MBC was determined.

#### 2.4.3. Effects of p(DH)@CHX on reducing S. mutans biofilm

As is demonstrated, 156-μg/mL and 78-μg/mL p(DH)@CHX could reduce the toxicity of CHX; 156-μg/mL and 78-μg/mL of p(DH)@CHX, 25-μg/mL and 12.5-μg/mL CHX were used to treat *S. mutans* biofilm. To evaluate the antibiofilm properties, *S. mutans* biofilms were incubated on glass disks in BHIS and incubated at 5% CO_2_ and 37 °C, as described before. After 24 hours, the biofilms were treated with p(DH)@CHX and CHX for one hour; then, lactic acid production measurement, MTT assay, CFU counting, SEM observation, and live/dead bacterial staining were conducted.

##### 2.4.3.1. LDH measurement

The disks (*n* = 6) with *S. mutans* biofilms were rinsed in cysteine peptone water. Each disk was placed in a new 24-well plate with 1.5 mL of buffered peptone water (BPW) supplemented with 0.2% sucrose. The disks with biofilms were incubated at 5% CO_2_ and 37 °C for three hours to allow the biofilms to produce acid. An enzymatic (lactate dehydrogenase) method was used to determine lactate concentrations in the BPW solutions (Wang et al., 2014). A microplate reader (SpectraMax M5; Molecular Devices, Sunnyvale, CA) was used to measure the absorbance at 340 nm for the collected BPW solutions. Standard curves were prepared using standard lactic acid (Supelco Analytical, Bellefonte, PA).

##### 2.4.3.2. MTT assay

The MTT (3-(4,5-dimethyl-thiazol-2-yl)-2,5-diphenyltetrazolium bromide) assay was conducted according to a previous study (Wang et al., 2016). Furthermore, 1 mL of MTT dye (0.5-mg/mL MTT in PBS) was incorporated into each well of the 24-well plate with the 48-hour biofilms on the disks. The plate was cultured at 37 °C anaerobically for an hour. Then, the disks were transferred to a new 24-well plate with 1 mL of dimethyl sulfoxide (DMSO) to dissolve the formazan crystals by shaking horizontally at 80 rpm for 20 minutes at room temperature in the dark. After being pipetted, 200 μL of the DMSO solution from each well was added to a 96-well plate, and the absorbance was measured at the OD_540nm_ via a microplate reader.

##### 2.4.3.3. CFU counts

To confirm biofilm inhibition, we performed viable counts on biofilm cells. The treated biofilms were washed three times with PBS. The biofilms were then resuspended in 300 μL of PBS, pipetted vigorously for 60 seconds (to disrupt the biofilms), serially diluted, and plated on BHI agar plates. CFUs were counted after overnight incubation at 37 °C (Zhou et al., 2016).

##### 2.4.3.4. SEM observation

The biofilms on the disks were rinsed with PBS and then immersed in 1% glutaraldehyde in PBS for four hours at 4 °C. The specimens were rinsed with PBS, subjected to a graded ethanol dehydration procedure, and rinsed twice with 100% hexamethyldisilazane. The specimens were then sputter-coated with gold and examined via scanning electron microscopy (SEM, Quanta 200, FEI, Hillsboro, OR) (Wang et al., 2016).

##### 2.4.3.5. Live/dead staining

As for the live/dead staining, the biofilms on the glass disks were washed three times with PBS and then stained using the BacLight live/dead bacterial viability kit (Molecular Probes, Eugene, OR). Live bacteria were stained with Syto9 to produce green fluorescence, and bacteria with compromised membranes were stained with propidium iodide to produce red fluorescence. The disks were examined under a confocal laser scanning microscope (CLSM) (Olympus FV3000, Tokyo, Japan). All the tests were repeated three times (Kumar Tiwari et al., 2019).

In addition, *S. mutans* biofilms were treated with p(DH)@CHX and CHX in neutral environments, too. For pH control, *S. mutans* biofilms were incubated in BHI broth supplemented with 1% sucrose and 50-mM PIPES (BHIS + PIPES, pH = 7.0) (Liang et al., 2020). The lactic acid production measurement and MTT assay were conducted to assess the viability of the biofilms. The methods were the same as described previously.

### 2.5. Effect on microcosm biofilm

To confirm the effect of p(DH)@CHX on microcosm biofilm, MTT assay and LDH measurements were used to examine the effect on the growth and metabolism of healthy saliva-derived biofilms, and 16S rRNA gene sequencing was used to determine bacterial diversity and community.

#### 2.5.1. Saliva collection and dental plaque microcosm biofilm model

As for the saliva collection, this study was authorized by the Ethics Committee of West China School of Stomatology, Sichuan University (Chengdu, China, WCHSIRB-D-2020-382). Ten healthy individuals without active caries or periodontal disease and with natural dentition provided healthy salivary samples. Donors did not take any antibiotics three months before the study and fasted for two hours before sample collection (Cheng et al., 2012). Moreover, the donors gargled with water before salivary samples were collected. The saliva was pooled and diluted two folds with 50% sterile glycerol. Then the salivary samples were stored at −80 °C (Cheng et al., 2011).

A McBain artificial saliva growth medium was used for all the biofilm experiments. This medium contained 2.5 g/L of type II mucin (porcine, gastric, Millipore Sigma, St. Louis, MO), 2.0 g/L of bacteriological peptone (Becton Dickinson, Sparks, MD), 2.0 g/L of tryptone (Becton Dickinson, Sparks, MD), 0.35 g/L of NaCl, 1.0 g/L of yeast extract (Fisher Scientific, Waltham, MA), 0.2 g/L of potassium chloride (Millipore Sigma, St. Louis, MO), 0.1 g/L of cysteine hydrochloride (Millipore Sigma, St. Louis, MO), 0.2 g/L of calcium chloride (Millipore Sigma, St. Louis, MO), and 1% sucrose (Solarbio, Beijing, CN). The pH of the medium was adjusted to 7, and 50-mM PIPES was added to the medium. After autoclaving, 0.0002 g/L of vitamin K1 and 0.001 g/L of hemin were added (Mitwalli et al., 2020).

#### 2.5.2. LDH measurements and MTT assay

Each sterile glass disk was transferred into a well of a polystyrene 24-well flat-bottomed microtiter plate, after which 1.5 mL of McBain medium was added. The saliva-glycerol stock was seeded (1:50 final dilution) into plates and incubated at 37 °C for 24 hours under anaerobic conditions. The medium was refreshed every 12 hours. After 24 hours, 156 μg/mL of p(DH)@CHX and 25 μg/mL of CHX were used to treat the biofilms for one hour. Then, PBS was used to rinse the biofilms to remove loose bacteria before immersing in a fresh medium (Huang et al., 2019). The procedures of lactic acid production measurement and MTT assay are described in Section 2.4.3.

##### 2.5.3. 16s rDNA sequencing analysis

16s rRNA sequencing was used to investigate the ecological impact of p(DH)@CHX and CHX on saliva-derived biofilms. The DNA was first extracted from the saliva-derived biofilms by the E.Z.N.A.^®^ Soil DNA Kit (Omega Bio-tek, Norcross, GA). Nanodrop (Thermo Scientific, Wilmington, NC) and agarose gel electrophoresis were used to assess the DNA concentration and quality, respectively. The universal target V4–V5 regions of the 16S rRNA gene were PCR-amplified using barcoded primers 515 F (5′-GTGCCAGCMGCCGCGG-3′) and 907 R (5nodrop; Thermo Scientific, Wilmington, NC), PCR reactions were performed in triplicate with 20 µL of the mixture containing 4 μL of 5× FastPfu Buffer, 2 μL of 2.5-mM dNTPs, 0.8 μL of each primer (5 μM), 0.4 μL of FastPfu polymerase, and 10 ng of template DNA. The amplicons were then extracted from 2% agarose gels and further purified using the AxyPrep DNA Gel Extraction Kit (Axygen Biosciences, Union City, CA) and quantified by QuantiFluor-ST (Promega, Madison, WI) according to the protocols. The amplicons were sequenced by the PacBio Sequel platform (Shanghai Personal Biotechnology Co., Ltd., Shanghai, China). An average of 5000 reads per sample was generated from the amplicon library. Sequence data analyses were mainly performed using QIIME (v1.8.0) and R packages (v3.2.0), including quality control of raw data and taxonomic annotation according to the NCBI database (Wang et al., 2016; Du et al., 2021).

### 2.6. Statistical analysis

The experimental results were analyzed with SPSS 22.0 (SPSS Inc., Chicago, IL). ANOVA with post hoc Tukey multiple comparisons was applied for multiple group comparisons. A standard *t*-test was used to analyze the data between the two groups. *p*<.05 indicated a significant difference. All the experiments were repeated at least three times.

## 3. Results

### 3.1. Characterization of poly(DMAEMA-co-HEMA)

**Figure 2.**
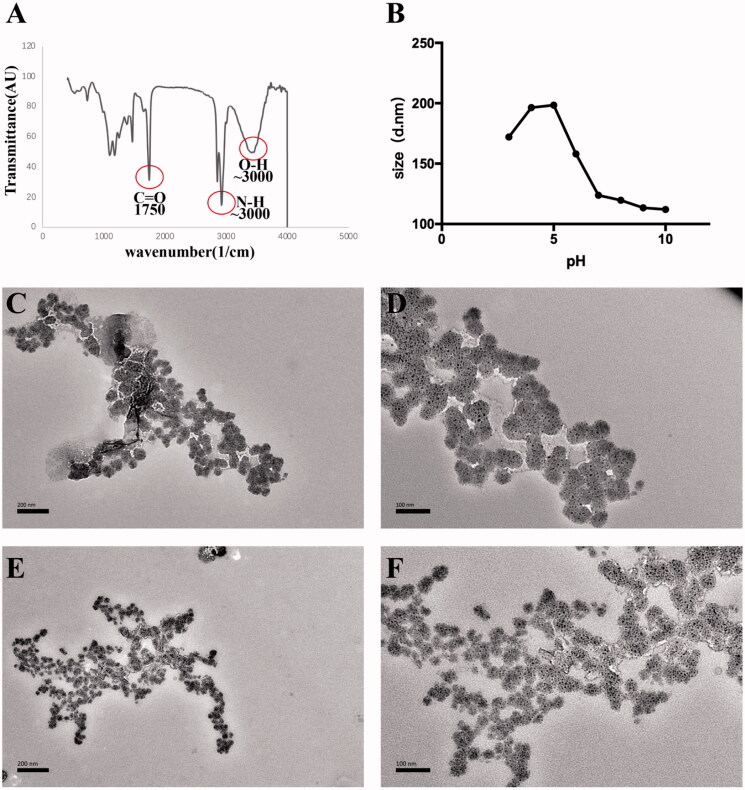
Characterization of poly(DMAEMA-co-HEMA). (A) FT-IR spectrum of poly(DMAEMA-co-HEMA). (B) Size of poly(DMAEMA-co-HEMA) at different pH values of 3, 4, 5, 6, 7, 8, 9, and 10. (C) TEM imaging of poly(DMAEMA-co-HEMA) at pH = 4.5 (×20,000 magnification). (D) TEM imaging of poly(DMAEMA-co-HEMA) at pH = 4.5 (×40,000 magnification). (E) TEM imaging of poly(DMAEMA-co-HEMA) at pH = 7 (×20,000 magnification). (F) TEM imaging of poly(DMAEMA-co-HEMA) at pH = 7 (×40,000 magnification).

According to the FT-IR results, poly(DMAEMA-co-HEMA) synthesis was performed successfully using two elements, including HEMA, DMAEMA, and RAFT polymerization methods. The functional groups were observed in the FT-IR spectrum as expected, representing the accomplishment of the polymerization steps (Figure 2(A)). The obtained peaks in the FT-IR spectrum represented the exact location of the existing groups, including (∼3000 cm^−1^), indicating N–H groups of DMAEMA (∼3500 cm^−1^), indicating O–H groups of HEMA in the spectrum, and (1670–1820 cm^−1^), indicating the presence of C=O groups in all the monomers. Thus, the target functional polymers were successfully synthesized according to the characterization.

The size of poly(DMAEMA-co-HEMA) was detected at different pH values. As shown in Figure 2(B), the particle sizes of the carrier were different under different pH conditions. The maximum particle size was around 200 nm at a pH of 4 or 5. In contrast, the average size of poly(DMAEMA-co-HEMA) at neutral pH (i.e. 7) was <150 nm. Moreover, the size of the carrier with two different pH values (4.5 and 7) was observed by TEM. In Figure 2(C–F), the particle size of poly(DMAEMA-co-HEMA) in the acid environment (pH = 4.5) is significantly larger than its particle size in the neutral environment (pH 7), consistent with the above results. Hence, the particle size was bigger in the acidic environment, corresponding to the cariogenic environment.

### 3.2. Drug loading and *in vitro* CHX release from p(DH)@CHX

The amount of the loaded drug was calculated by measuring the amount of CHX that was not encapsulated in the carrier. When 7.5 mg of CHX was added to 37.5 mg of poly(DMAEMA-co-HEMA), the CHX loading capacity and encapsulation efficiencies of poly(DMAEMA-co-HEMA) were calculated at 16.03% and 80.15%, respectively (Table 1).

**Table 1.** Drug loading efficiency and drug loading amount of poly(DMAEMA-co-HEMA).

CHX amount (mg)	Carrier amount (mg)	DLC (%)	EE (%)
7.5	37.5	16.03 ± 1.3	80.15 ± 6.5

EE: encapsulation efficiency; DLC: drug loading capacity.

Data are mean ± SD, *n* = 3.

**Figure 3.**
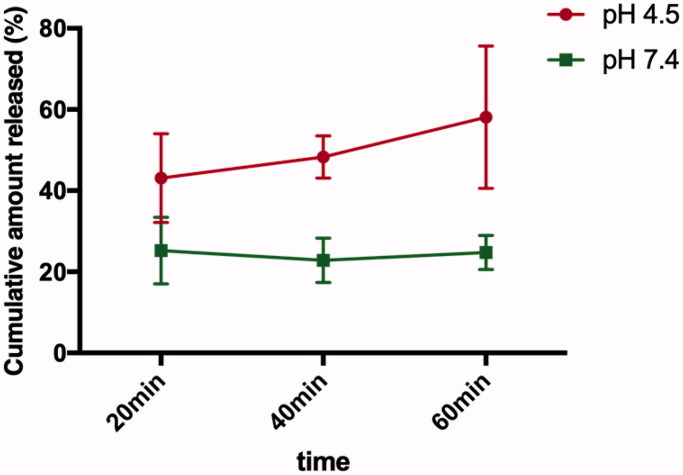
Release profiles of p(DH)@CHX. The pH-responsive release profiles of CHX from p(DH)@CHX in PBS solution of pH values of 4.5 and 7.

The *in vitro* release behavior of CHX from p(DH)@CHX was evaluated at different pH values (7 and 4.5). Within one hour, the amount of CHX released in the acidic environment was significantly higher than that in the neutral environment. At the neutral pH, the release of CHX remained stable over time, while at the acidic pH, the release of CHX increased gradually. After 60 minutes, the cumulative amount of CHX in the acidic environment reached 60% (Figure 3).

### 3.3. Evaluation of cytotoxicity of poly(DMAEMA-co-HEMA) and p(DH)@CHX

**Figure 4.**
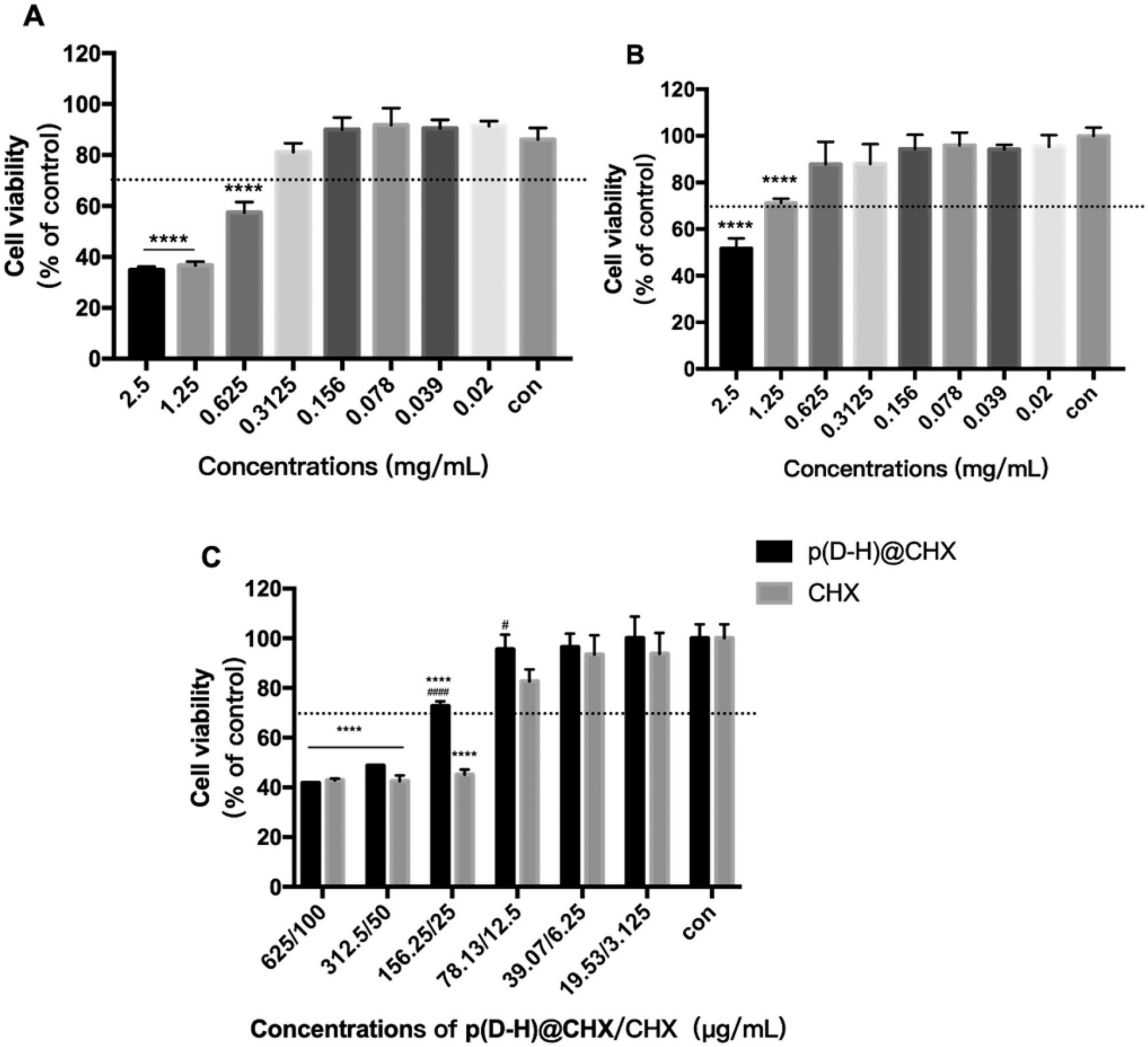
Cell cytotoxicity assay. (A) CCK-8 assay of poly(DMAEMA-co-HEMA) at pH = 4.5. (B) CCK-8 assay of poly(DMAEMA-co-HEMA) at pH = 7. (C) CCK-8 assay of p(DH)@CHX and free CHX. Data are presented as means ± standard deviations (****p<.0001 vs. the control group, ^#^*p*<.05 vs. the CHX group, ^####^*p*<.0001 vs. the CHX group).

HOK cells were exposed to culture media with different concentrations of poly(DMAEMA-co-HEMA) in environments with different pH values for one hour, and the cell viability was evaluated to verify the feasibility of the pH-responsive nanocarrier system (Figure 4(A,B)). As shown in Figure 4(A), at an acidic pH (i.e. 4.5), the relative cell viability reached 70% of the control when the concentration of poly(DMAEMA-co-HEMA) was <0.3125 mg/mL. In addition, as shown in Figure 4(B), under the neutral pH at 1.25 mg/mL, the cell survival rate of poly(DMAEMA-co-HEMA) was still 70%. Taken together, under neutral or acidic conditions, when the concentration was <0.3125 mg/mL, poly(DMAEMA-co-HEMA) exhibited low cell toxicity and good biocompatibility.

After drug loading, the cytotoxicity of p(DH)@CHX and free CHX was also evaluated (Figure 4(C)). p(DH)@CHX did not exhibit significant cytotoxicity at concentrations <156.25 μg/mL, while free CHX showed ∼45% cell viability at a corresponding concentration of 25 μg/mL. In addition, 156.25-μg/mL and 78.13-μg/mL p(DH)@CHX showed higher cell activity than corresponding concentrations (25 μg/mL and 12.5 μg/mL) of CHX, and the difference was statistically significant (156.25 μg/mL: *p*<.0001; 78.13 μg/mL: *p*<.05). It is well established that high doses of CHX can trigger oral cell death, resulting in unwanted side effects (Müller et al., 2017). We demonstrated that p(DH)@CHX with a stimuli-responsive drug release behavior could reduce CHX toxicity to normal cells. These findings provide a concentration reference for subsequent research.

### 3.4. Antibacterial and anti-biofilm effects on *S. mutans*

**Figure 5.**
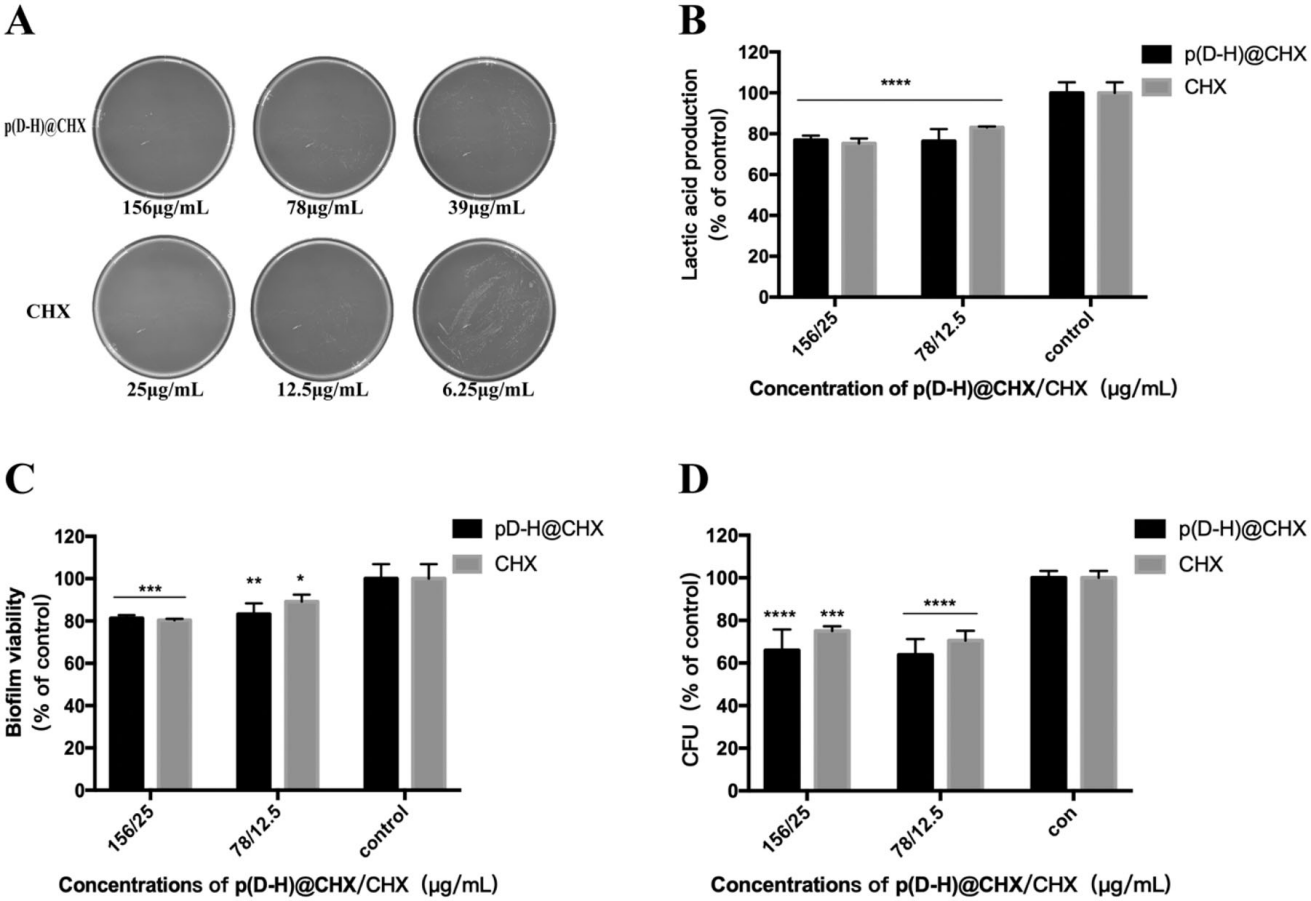
Antibacterial effect on the development of *S. mutans* biofilm. (A) Photographs of BHI agar plates coated with *S. mutans* supplemented with different concentrations of p(DH)@CHX and free CHX. (B) Lactic acid production by *S. mutans* biofilms adhering to the disks. (C) The MTT metabolic activity in the three groups. (D) CFU counts of *S. mutans* biofilms adhering to the disks. Data are presented as means ± standard deviations (*p<.05, **p<.01, ***p<.001, ****p<.0001).

As *S. mutans* is the main etiologic factor for dental caries, we applied the *S. mutans* biofilm model to investigate the antibacterial activity of p(DH)@CHX. The MIC and MBC of p(DH)@CHX poly(DMAEMA-co-HEMA) and free CHX were determined for *S. mutans*, using broth microdilution. Table 2 summarizes the results. As expected, poly(DMAEMA-co-HEMA) showed no antimicrobial activity, and p(DH)@CHX completely inhibited bacterial growth at concentrations >9.77 μg/mL, demonstrating that the corresponding MIC of p(DH)@CHX was 9.77 μg/mL, while the MIC of free CHX was 1.56 μg/mL. In addition, the MBCs of p(DH)@CHX and free CHX against *S. mutans* were 156.25 μg/mL and 25 μg/mL, respectively, as shown in Figure 5(A). Overall, p(DH)@CHX and CHX exhibited good bacterial killing efficacy by testing their MIC and MBC against *S. mutans*, and p(DH)@CHX retained strong antimicrobial effects against the planktonic *S. mutans* of CHX.

**Table 2.** MIC and MBC values of *S. mutans* for the components used in this study.

	MIC	MBC
p(DMAEMA-HEMA)@CHX	9.77 μg/mL	156.25 μg/mL
CHX	1.56 μg/mL	25 μg/mL
p(DMAEMA-HEMA)	No effect	–

*S. mutans* biofilms were treated by different concentrations of p(DH)@CHX and CHX to further investigate the antibacterial effects of p(DH)@CHX in biofilms. We treated cariogenic biofilms for one hour to verify the fast response and short-term anti-biofilm effect of p(DH)@CHX. The lactic acid production by biofilms is plotted in Figure 5(B). The biofilms in the control group produced the most lactic acid. Different concentrations p(DH)@CHX and free CHX all reduced acid production, compared to the control (*p< .0001). In addition, there was no statistically significant difference in lactic acid production between the p(DH)@CHX groups and CHX groups (*p>.05). The biofilm metabolic activity was measured using the MTT assay, with the results plotted in Figure 5(C). At all the experimental groups, p(DH)@CHX and CHX reduced the metabolic activity of *S. mutans* biofilm compared to the control group (156-μg/mL p(DH)@CHX group and 25-μg/mL CHX group; *p<.001; 78-μg/mL p(DMAEMA-HEMA)@CHX group: *p<.01; 12.5-μg/mL CHX group: *p<.05). However, there was no statistically significant difference between the p(DH)@CHX groups and the corresponding CHX groups (*p>.05). The CFU counts were determined on the treated biofilms to measure the biomass of biofilms. As shown in Figure 5(D), there was no significant difference between p(DH)@CHX groups and the corresponding CHX groups in CFU counts (*p>.05). In addition, compared to the control group, the CFU counts decreased after being treated with p(DH)@CHX or CHX, with significant differences (25-μg/mL CHX group: *p<.001, others: *p<.0001).

**Figure 6.**
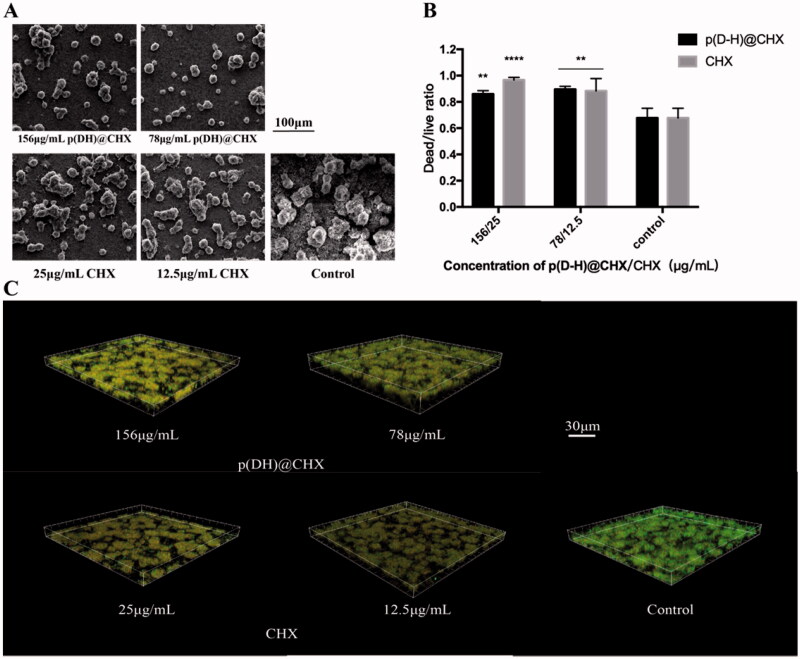
(A) Scanning electron microscope (SEM) micrographs (×1000). (B) The dead/live cell ratios for the three groups. The results were averaged from three randomly selected views of each group. (C) Live/dead staining of biofilms on the cured disks of the three groups. Live cells were stained green, and dead cells were stained red. The data are presented as means ± standard deviations (**p<.01, ****p<.0001).

Figure 6(A) presents the SEM images of each group. The disks in the control group had dense biofilms compared with the other two groups. The p(DH)@CHX and CHX groups had fewer bacteria in the biofilm than the control group. The biofilm quantities in the p(DH)@CHX groups were only slightly less than those in the CHX groups, visible under a microscope, but the differences were not noticeable. Figure 6(B) shows the live/dead bacterial staining of the biofilms in different groups. Live bacteria were stained green, and dead bacteria were stained red. More live cells could be detected in the biofilms from the control group than those from the p(DH)@CHX and free CHX groups. The dead/live bacteria ratios were computed with three randomly selected views of each group, as plotted in Figure 6(C). Free CHX and loaded CHX exhibited reduced biofilms on the surface and increased proportion of dead bacteria in the biofilm compared with the control group, with statistically significant differences (25-μg/mL CHX group: *p*<.0001, other groups: *p*<.01).

**Figure 7.**
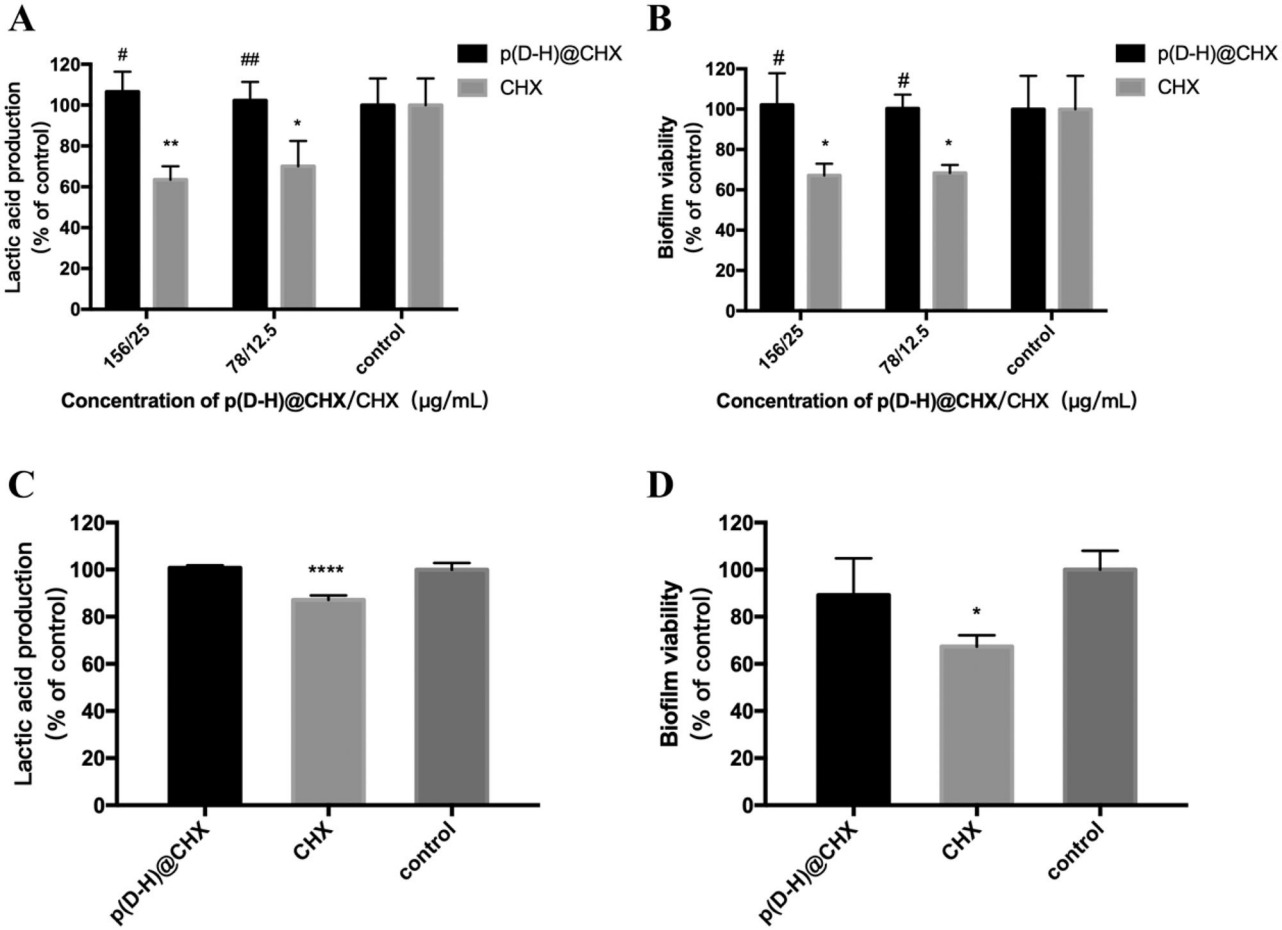
(A) Lactic acid production by *S. mutans* biofilm after treating with p(DH)@CHX or CHX at pH = 7. (B) MTT metabolic activity of *S. mutans* biofilm after treating with p(DH)@CHX or CHX at pH = 7. (C) Lactic acid production by healthy saliva-derived biofilms after treating with p(DH)@CHX or CHX at pH = 7. (D) MTT metabolic activity of healthy saliva-derived biofilms after treating with p(DH)@CHX or CHX at pH = 7. Data are presented as mean ± standard deviation (*p<.05, **p<.01, ****p<.0001 vs. the control group, ^#^*p*<.05, ^##^*p*<.01 vs. the CHX group).

Furthermore, we explored the antimicrobial effect of p(DH)@CHX on *S. mutans* biofilm in a neutral environment. Figure 7(A) shows lactic acid production of biofilms. Compared with the control group, the 156-μg/mL and 78-μg/mL p(DH)@CHX groups exhibited no significant differences (*p*> .05). In contrast, the lactic acid production of the 25-μg/mL free CHX group decreased by ∼60% compared to the control group (*p*< .01), and the 12.5-μg/mL free CHX group exhibited reduced lactic acid production by ∼70% compared to the control group (*p*< .05). Also, the differences between the free CHX and the drug-loaded groups were significant. The MTT results are plotted in Figure 7(B). As in the acidic environment, the free CHX groups exhibited significantly decreased biofilm metabolic activity compared with the control group (*p*< .05), while no significant differences were found between the p(DH)@CHX groups and the control group (*p*>.05). Taken together, p(DH)@CHX did not exert the antibacterial effect of CHX in the neutral environment and could not inhibit the growth of *S. mutans* biofilm.

### 3.5. Effect on microcosm biofilm

To thoroughly investigate the effect of p(DH)@CHX on microcosm biofilm, MTT assay and LDH measurement were used to examine the effect on the growth and metabolism of healthy saliva-derived biofilm, and 16S rRNA gene sequencing was used to determine bacterial diversity and community. The MTT assay and lactic acid production results are plotted in Figure 7(C,D). Compared with the control group, the lactic acid production of the 25-μg/mL free CHX group decreased slightly by approximately 12.84% (*p*<.0001). However, 156-μg/mL p(DH)@CHX did not affect lactic acid production of healthy saliva-derived biofilms, with no significant difference from the control group (*p*>.05). The MTT results are plotted in Figure 7(D). The control group and 156-μg/mL p(DH)@CHX group exhibited similar metabolic activity (*p*>.05). The 25-μg/mL free CHX group decreased the biofilm metabolic activity by approximately 32.7% (*p*<.05). Thus, p(DH)@CHX showed no antimicrobial effect on saliva-derived microcosm biofilm.

**Figure 8.**
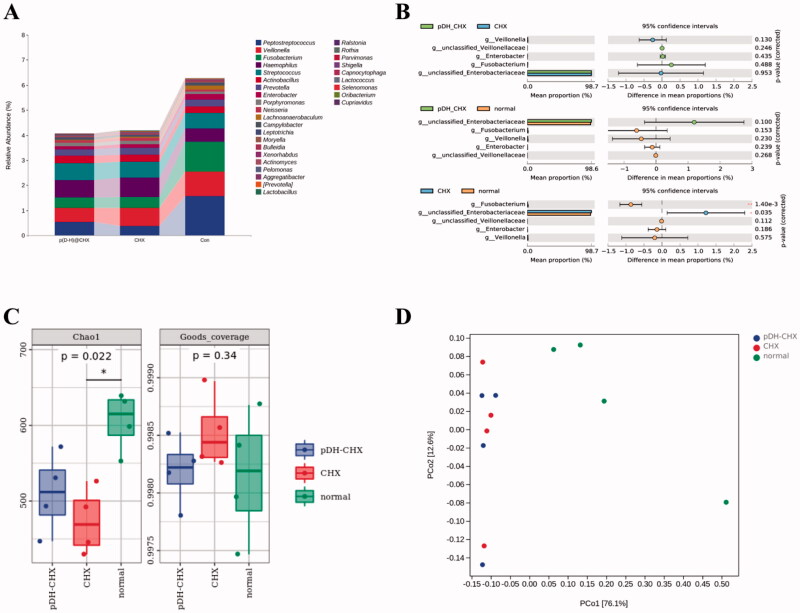
The microbial community of saliva-derived biofilms. (A) The percentage of community abundance at the genus level. (B) The comparison of several genera among the three groups. (C) The alpha diversity of all the groups was measured using the Chao1 index and Good’s average index. (D) A principal coordinates analysis (PCoA) score plot of the three groups.

Figure 8(A–D) presents the 16S rRNA gene sequencing data. Figure 8(A) presents the percentage of community abundance at the genus level. As we can see, there was a higher percentage of *Peptostreptococcus*, *Veillonella*, *Fusobacterium*, *Haemophilus*, *Streptococcus*, and *Prevotella* in all the groups. Then, several genera with noticeable differences were analyzed individually, with the results plotted in Figure 8(B). Compared with the control group, there was decreased abundance of *Peptostreptococcus* in the p(DH)@CHX and CHX groups, while the abundance in the p(DH)@CHX group was slightly higher than in the CHX group. Moreover, the abundances of *Veillonella*, *Fusobacterium*, *Actinobacillus*, and *Enterobacter* were the highest in the control group, with the lowest in the p(DH)@CHX group. As shown in Figure 8(C), alpha diversity was calculated using the Chao1 indexes and Good’s coverage index. The Chao1 index was used to estimate species richness. Compared with the control group, the CHX group had a lower Chao1 richness (*p*<.05), while the p(DH)@CHX group had a slightly higher Chao1 richness. However, the difference was not statistically significant (*p*>.05), indicating that the species richness of healthy saliva-derived biofilm decreased after treatment with free CHX and that p(DH)@CHX had no significant effect on the species richness of the biofilm. Also, Good’s coverage index was used to evaluate whether the sequencing depth was adequate for diversity analysis, which was >99.7% for all the samples. In addition, the principal coordinates analysis (PCoA) showed that the biofilms of the three groups were distinctly separate from one another (Figure 8(D)).

## 4. Discussion

It is well established that bacteria are closely related to dental caries. Previous studies have shown that the application of antimicrobial drugs has a caries-preventive effect. However, traditional agents have been associated with many drawbacks in previous studies. In recent decades, novel intelligent materials have become a research hotspot gradually (Horev et al., 2015; Liang et al., 2020). In this study, a well-established drug carrier, poly(DMAEMA-co-HEMA), was synthesized. Poly(DMAEMA-co-HEMA) is responsive to the environment pH and has been widely employed in the targeted delivery of various anti-cancer agents such as paclitaxel, 5-FU, etc. (You & Auguste, 2008; Roointan et al., 2018). We first used poly(DMAEMA-co-HEMA) to load CHX, a commonly used antibacterial agent to inhibit dental biofilm formation, to prevent dental caries in clinical practice. FT-IR, DLS, and TEM results showed the successful synthesis of poly(DMAEMA-co-HEMA) with good pH-sensitive properties. Then, we constructed and successfully prepared CHX-loaded p(DH)@CHX nanocomposites and confirmed that the nanoparticle had good drug-loading, pH-sensitive *in vitro* release, and biocompatibility at different pH values. In addition, we observed that p(DH)@CHX significantly reduced the toxicity of free CHX, which might solve this problem in the clinical application of CHX.

As the primary etiologic factor of dental caries, *S. mutans* was an ideal target for preventing and treating dental caries, and it was used in most caries prevention studies (Lemos et al., 2019). Also, *S. mutans* was the main candidate to form acidic environments; therefore, we used it as a biofilm model to investigate the antibacterial activity of p(DH)@CHX (Scharnow et al., 2019). In this research, we simultaneously evaluated the antibiotic effect of p(DH)@CHX and free CHX on planktonic and biofilm states. According to the results of the tests to detect *S. mutans* planktonic bacteria and biofilms, p(DH)@CHX could exert the antimicrobial activity of CHX. Thus, to some extent, p(DH)@CHX may play a protective role against dental caries. At the same time, the anti-biofilm experiment in a neutral environment showed that p(DH)@CHX only functioned in an acidic cariogenic environment. Therefore, p(DH)@CHX can retain the antimicrobial effect of free CHX while reducing its toxicity. However, the antibiofilm effect of p(DH)@CHX is not excellent. It is still necessary to develop more effective and practical methods to overcome the shortcomings of the existing methods, increase drug loading efficiency, and enhance the adhesion to the biofilm.

Oral health and disease are correlated with the interplay within the oral microbial community (Simon-Soro et al., 2018). According to the ecological plaque hypothesis, many microbial communities maintain a dynamic balance with the host organism in normal conditions (Burne, 2018; Rosier et al., 2018). Changes in the environment can lead to a shift in the microbiota where aciduric and acidogenic species proliferate, ultimately leading to carious lesions (Reyes et al., 2014; Duran-Pinedo & Frias-Lopez, 2015). Nowadays, the therapy of dental caries is associated with a series of serious challenges. For example, CHX has a pernicious effect on both healthy biofilms in a balanced state and cariogenic biofilms in an unbalanced state in the oral cavity, which is not good for the oral microecological balance (Marsh, 1994). Poly(DMAMEA-co-HEMA), as a pH-sensitive carrier, after loading CHX, can only release some CHX in a neutral environment. To further evaluate the effect of p(DH)@CHX on healthy saliva-derived biofilms, we conducted *in vitro* antibiofilm tests and 16s rDNA sequencing experiments. The results showed that p(DH)@CHX would not impact healthy saliva-derived biofilms when used in the oral environment, avoiding the disruption of the microecological balance. We used 16s rDNA sequencing for microbial community analysis to identify the microbiome of healthy saliva-derived biofilms. As shown in Figure 8(B), the abundance of *Peptostreptococcus* in the p(DH)@CHX and the CHX groups was less than the control group, while the abundance in the p(DH)@CHX group was slightly higher than in the CHX group. It is worth noting that *Peptostreptococcus* is a member of the normal flora of the human oral cavity, contributing to its functional stability and microecological balance and health (Sizova et al., 2015). Therefore, we can infer that p(DH)@CHX can reduce the disruption of the microecological balance of CHX. In addition, *Veillonella*, *Fusobacterium*, *Actinobacillus*, and *Enterobacter* accounted for most of the microorganisms in the control group and the least in the p(DH)@CHX group. A previous study demonstrated that *V. parvula* might participate in caries development through interactions with *S. mutans* (Liu et al., 2020). Also, it was reported that *Enterobacter* was one of the main cariogenic genera and played a role in inducing dental caries (Goldberg et al., 1997; Davis et al., 2005; Wang et al., 2021). We can predict that p(DH)@CHX maintained the caries-prevention effect but not the microecological balance. Furthermore, *F. nucleatum* and *A. actinomycetemcomitans* are periodontal pathogenic bacteria. Among them, *A. actinomycetemcomitans* is the main pathogenic bacteria of aggressive periodontitis (Velliyagounder et al., 2012; Azzimonti et al., 2015; Kim et al., 2020), and *F. nucleatum* is the main pathogenic bacteria responsible for acute necrotizing ulcerative gingivitis (Tefiku et al., 2020). Thus, it indicated the potential of p(DH)@CHX for the prevention and treatment of periodontal diseases.

Collectively, p(DH)@CHX, as a novel ‘intelligent material,’ could minimize the cytotoxicity of CHX and inhibit the caries-associated biofilms without destroying normal oral flora. It exhibited the potential to be used for caries prevention and treatment. Thus, we envision that p(DH)@CHX can be used like the fluorinated foam, which has a tray and can keep the formulation in close contact with the teeth.

Furthermore, as MMPs also play a critical role in dentinal caries progression, they degrade collagen in carious lesions. In addition, MMPs responsive drug delivery systems have been widely used in cancer therapy. Therefore, we can combine this with pH-sensitive drug delivery systems to design a dual-sensitive system. We plan to work toward this goal as the next step in our research.

## 5. Conclusions

In conclusion, we synthesized a well-studied pH-sensitive carrier poly(DMAEMA-co-HEMA) and then loaded CHX for the first time. The novel drug delivery system can inhibit the development of *S. mutans* biofilm and regulate the oral microecosystem. Therefore, it might target the cariogenic biofilm and prevent dental caries incidence. Furthermore, this research has also provided a potential method for caries prevention.

## Data Availability

The authors confirm that the data supporting the findings of this study are available within the article.
